# Nontherapeutic equivalence of a generic product of imipenem-cilastatin is caused more by chemical instability of the active pharmaceutical ingredient (imipenem) than by its substandard amount of cilastatin

**DOI:** 10.1371/journal.pone.0211096

**Published:** 2019-02-06

**Authors:** Maria Agudelo, Carlos A. Rodriguez, Andres F. Zuluaga, Omar Vesga

**Affiliations:** 1 GRIPE: *Grupo Investigador de Problemas en Enfermedades Infecciosas*, University of Antioquia (UdeA) Medical School, Medellín, Colombia; 2 Infectious Diseases Unit, *Hospital Universitario San Vicente Fundación*, Medellín, Colombia; 3 CIEMTO: *Centro de Información de Medicamentos y Tóxicos*, University of Antioquia (UdeA) Medical School, Medellín, Colombia; Tallinn University of Technology, ESTONIA

## Abstract

**Background:**

We demonstrated therapeutic nonequivalence of “bioequivalent” generics for meropenem, but there is no data with generics of other carbapenems.

**Methods:**

One generic product of imipenem-cilastatin was compared with the innovator in terms of in vitro susceptibility testing, pharmaceutical equivalence, pharmacokinetic (PK) and pharmacodynamic (PD) equivalence in the neutropenic mouse thigh, lung and brain infection models. Both pharmaceutical forms were then subjected to analytical chemistry assays (LC/MS).

**Results and conclusion:**

The generic product had 30% lower concentration of cilastatin compared with the innovator of imipenem-cilastatin. Regarding the active pharmaceutical ingredient (imipenem), we found no differences in MIC, MBC, concentration or potency or AUC, confirming equivalence in terms of in vitro activity. However, the generic failed therapeutic equivalence in all three animal models. Its *E*_*max*_ against *S*. *aureus* in the thigh model was consistently lower, killing from 0.1 to 7.3 million less microorganisms per gram in 24 hours than the innovator (P = 0.003). Against *K*. *pneumoniae* in the lung model, the generic exhibited a conspicuous Eagle effect fitting a Gaussian equation instead of the expected sigmoid curve of the Hill model. In the brain infection model with *P*. *aeruginosa*, the generic failed when bacterial growth was >4 log_10_ CFU/g in 24 hours, but not if it was less than 2.5 log_10_ CFU/g. These large differences in the PD profile cannot be explained by the lower concentration of cilastatin, and rather suggested a failure attributable to the imipenem constituent of the generic product. Analytical chemistry assays confirmed that, besides having 30% less cilastatin, the generic imipenem was more acidic, less stable, and exhibited four different degradation masses that were absent in the innovator.

## Introduction

The World Health Organization (WHO) and all Drug Regulatory Agencies (DRA) use the term “bioequivalence” to imply that a generic product has identical concentration and potency with respect to the innovator (pharmaceutical equivalence) and a similar pharmacokinetic profile (pharmacokinetic equivalence); from that it is assumed that both products have the same efficacy in vivo (therapeutic equivalence). However, there is substantial experimental evidence that the bioequivalence assumption is wrong [1–10]. Although bioequivalence does predict therapeutic equivalence of generic antimicrobials obtained by chemical synthesis like metronidazole [7], ciprofloxacin [8] and fluconazole [9], it does not for antibiotics *sensu stricto* (i.e., those obtain from microorganisms), like aminoglycosides [1, 5], penicillins [2, 6], and glycopeptides [3].

The manufacture of complex medicines requires knowledge unavailable to the makers of generic active pharmaceutical ingredients (API), and some compounds are definitely more difficult to imitate than others [10, 11]. The oversimplification of the process for marketing generics was adopted as an ideal model by the WHO and DRA to provide universal access to essential medicines with huge savings, and it did work out very well [12]. However, the data suggest that societies may have been paying a bigger price in terms of human health [13, 14], with incalculable consequences [6, 15, 16]. Therefore, research to determine the mechanisms by which a “bioequivalent” generic fails in vivo should be undertaken before the public trust in DRA is irreversibly eroded [17].

Carbapenems are among the last antibiotics still useful to combat infections caused by multidrug resistant pathogens [4, 18]. Therefore, it is of interest to establish if generic versions of imipenem, the first agent of its class to be approved, have any problem in terms of therapeutic equivalence. Here, we use an experimental approach to demonstrate how one generic product of imipenem-cilastatin, licensed in several nations to treat human patients, failed in vivo against wild-type (WT) and multi-drug resistant (MDR) bacterial pathogens encompassing Gram positive cocci, Enterobacteriaceae and non-fermenter Gram negative bacilli.

## Materials and methods

### 1. Bacteria, media and antibiotics

*Staphylococcus aureus* GRP-0057, *Klebsiella pneumonia*e GRP-0107, and *Pseudomonas aeruginosa* strains GRP-0019, GRP-0049, GRP-0036, and ATCC 27853 were used to infect the mice in the different models. The microorganisms were grown to log phase in Mueller-Hinton broth and agar for susceptibility tests, and in trypticase soy broth and agar for animal infection models (all from Becton Dickinson, Sparks, MD, USA). *Staphylococcus aureus* ATCC 29213, *K*. *pneumoniae* ATCC 43816 and *P*. *aeruginosa* 27853 were the quality control strains in susceptibility tests; *Kocuria rhizophila* ATCC 9341 was the seeding organism for microbiological assays. We bought the antibiotics from well-reputed local drugstores and reconstituted them following manufacturer instructions. The products, both licensed for human use by INVIMA (the Colombian DRA), included a generic product of imipenem (Inem, Ivax Pharmaceuticals, Mexico) and the innovator (Tienam, Merck Sharp & Dohme, Elkton, VA). Both products came in vials with a lyophilized powder containing imipenem 500 mg and cilastatin 500 mg; lot numbers and additional information about the products employed in this study are described in S1 Table.

### 2. In vitro experimentation

#### Susceptibility testing

Following CLSI methods [19], generic and innovator were compared in terms of their minimal inhibitory (MIC) and bactericidal (MBC) concentrations by broth microdilution (2–3 assays, each by duplicate) against *S*. *aureus* GRP-0057 and *K*. *pneumoniae* GRP-0107 (both WT isolates from patients with bacteremia), and against *P*. *aeruginosa* reference strain ATCC 27853 (WT) and *P*. *aeruginosa* clinical strains GRP-0019 (WT), GRP-0049 (MDR, susceptible to carbapenems) and GRP-0036 (MDR, carbapenem-resistant). The statistical significance of the difference in geometric means of MIC and MBC was determined with the Mann-Whitney U test.

#### Microbiological assay

The pharmaceutical equivalence of the generic product with respect to the innovator of imipenem was determined by comparing their standard curves obtained with a previously validated application of the microbiological assay [20]. Both drugs were tested simultaneously in a 36×36 cm plate originally described by Bennett in 1966 [21]; *K*. *rhizophila* ATCC 9341 was the agar seeding organism in Difco Antibiotic Media No. 8. Ten concentrations of imipenem were plated, each one was repeated 12 times, incubated for 18 h at 37°C under an aerobic atmosphere, and the same researcher measured all zone diameters with an electronic caliper. The standard curves were obtained by linear regression of log-transformed concentrations (log_10_ mg/L) plotted against their respective inhibition zones in millimeters (mean diameter and standard deviation of 12 inhibition zones per concentration). The linear regression parameters slope and intercept were then compared by curve-fitting analysis (CFA) with Prism 5 (GraphPad Software, Inc., La Jolla, CA). Pharmaceutical equivalence of the imipenem component was met when the regression lines of generic and innovator were parallel and overlaid, without significant difference in terms of the potency of the API (slope) or its concentration (intercept). Significant differences in one or both of these parameters implied a lack of pharmaceutical equivalence [22]. To determine pharmaceutical equivalence of the cilastatin component, we used HPLC-UV.

#### Standard curves by HPLC-UV: Apparatus, solutions and chromatographic conditions

For sample preparation, generic and innovator imipenem-cilastatin products were used for method development in the HPLC-UV. For the standard curves, imipenem Sigma was used as external standard. Methanol (MeOH) and HPLC-grade water were used to do the respective dilutions in the optimization conditions [23]. The high-pressure liquid chromatographic system consisted on a HPLC quaternary pump HP1100 instrument column oven (Hewlett-Packard, Waldbronn, Germany) that was used and connected to an UV detector of the same series. Separation was achieved at 20°C using a satisfaction C18 Luna column (250 mm × 4.6 mm, 5 μm). Mobile phase consisted of a mixture of buffer phosphate 0.1 M (pH 6.8–7.0) and acetonitrile in a 90:10 volume ratio delivered at 0.5 mL/min in an isocratic elution. Integration of peak areas and height for the data analysis was performed using the own software integrator. Each sample was left running during 15 min. The auto sampler temperature was kept at +8°C and the injection volume was 50 μL. The detection wavelength was set at 298 nm without attenuation in the absorbance units. All samples were assayed twice.

#### Stability in sterile physiologic saline solution

The effect of temperature on the stability of imipenem-cilastatin products was studied along 24 h. After product reconstitution, we monitored aliquots of imipenem-cilastatin spiked (2,500 and 5,000 mg/L) in sterile 0.9% saline solution for: (a) concentration and potency of the API immediately upon powder reconstitution -hour 0- and one day later -hour 24- standing at 4°C, 25°C and 37°C; (b) pH changes at 0, 6, 12, 18, and 24 h; and (c) color changes determined by spectrophotometric method at 0, 6, 12, 18, and 24 h. Semi logarithmic plots of API concentration, pH or optical density versus time were constructed to determine the rate and order of imipenem degradation.

#### Qualitative assay for mass spectrometry

Analytical chemistry data were obtained with an Agilent 1100 liquid chromatograph coupled to a mass spectrometer electrospray ionization VL system. At the stationary phase, we employed a Thermo Scientific Hypersil Gold analytical column (150 mm x 4.6 mm, 5 μm) for each product. We used the SIM mode to obtain the chromatogram and the SCAN mode to capture the mass spectra with a range of *m/z* 150–1000. The mobile phase consisted of A: 0.1% formic acid in water, B: 0.1% formic acid in acetonitrile (A 90:10 B); 0.5 mL/min as a flow rate and a total run time of 10 min. Working solutions for all studies were prepared by serial dilution of the stock solution (5,000 mg/L) [24]. To compare the chromatograms (SIM mode) and the mass spectra (SCAN mode), all preparations for reference material and pharmaceutical formulations were freshly prepared in deionized water at the moment of analysis using an imipenem-cilastatin concentration of 250 mg/L. The mobile phase was kept running in the equipment for 15 min prior to sampling and a blank sample was run after each product (performed at least twice).

#### Single-dose serum pharmacokinetics in neutropenic mice infected in the thighs with *P*. *aeruginosa* GRP-0019 (bioequivalence)

Imipenem generic product was studied simultaneously with the innovator at three dose levels, 10, 20 and 40 mg/kg. Two hours after infection, two groups of female mice (one for the innovator and one for the generic) received a single subcutaneous injection (0.2 mL) containing one of the three dose levels of imipenem to be tested. Data for each dose level and product were obtained from 9 mice divided in subgroups of 3 animals bled (100 μL by retro-orbital puncture) three times after dosing at 5, 45, 90 (first), 15, 60, 105 (second), and 30, 75, and 120 min (third subgroup). Serum was obtained by blood centrifugation at 10,000 g during 5 min and plated immediately (10 μL) in duplicate for microbiological assay. The parameters absorption rate constant (Ka), elimination rate constant (Ke), volume of distribution (V), first-order transfer rate constant from the central to peripheral compartment (KCP), and first-order transfer rate constant from the peripheral to central compartment (KPC) were obtained by population PK analysis with the nonparametric adaptive grid (NPAG) approach of the *Pmetrics* package (Laboratory of Applied Pharmacokinetics, University of Southern California; available at www.lapk.org) [25].

### 3. In vivo experimentation

#### Ethical considerations

The experimental protocol was reviewed and approved by the University of Antioquia Animal Experimentation Ethics Committee. Our lab and the institutional ethics committee define specific humane endpoints for any animal subjected to experimental procedures involving an infecting agent. For this study, animals were evaluated during 1 hour every 3 hours in search of the earliest clinical sign of impending death or poor prognosis of quality of life, or specific signs of severe suffering, pain or distress; therefore, the maximal delay for humane killing was 2 hours. By protocol, when animals reach any of these specific criteria, an experienced scientist must kill them immediately by cervical dislocation: (1) any mouse exhibiting extreme piloerection and lethargy, (2) any mouse hunched and incapable to move for drinking or to avoid manipulation, and (3) any mouse with seizures.

Since this study includes three different animal models of human infection, each model has some particularities regarding the welfare of the mice that we determined during the standardization of the models for previous projects [26]. The thigh model with *S*. *aureus* GRP-0057 has 0% lethality in untreated animals at 48 hours, and the model ends 26 hours after infection; none of 224 mice employed in this model ever reached criteria for humane killing (i.e., all animals were killed at the end of the experiment). For the pneumonia model with *K*. *pneumoniae* GRP-0107 we used 225 mice, death due to septic shock occurs between hours 36 and 60 in 100% of untreated animals, and the model ends 38 hours after infection (221 animals were humanely killed at the end of the model and 4 were found dead in their cages). The meningoencephalitis model with *P*. *aeruginosa* has a 100% lethality in untreated animals, mice die between hours 15 and 30 due to brain herniation, and the model ends 26 hours after infection; the mice started to die at hour 15 when inoculated with *P*. *aeruginosa* ATCC 27853, at hour 21 with strain GRP-0019, and at hour 27 with strain GRP-0049. For the meningoencephalitis model we used 324 mice, 12 were found dead in their cages, and 312 were humanely killed between hour 15 (60 mice) and the end of the experiments (252 mice).

#### Neutropenic mouse thigh infection model

Six-week old female MPF mice of the strain Udea:ICR(CD-1) weighing 23–27 g were immunosuppressed by intraperitoneal injections of cyclophosphamide (Endoxan, Baxter Oncology GmbH. D-33790 Halle, Germany) 4 days (150 mg/kg) and 1 day (100 mg/kg) before infection [26]. Sixteen hours after the second dose of cyclophosphamide, the animals were infected by inoculation of 5.03–5.30 log_10_ CFU of *S*. *aureus* GRP-0057 per thigh. Two experimental groups were designed to receive treatment with generic or innovator imipenem at daily doses ranging from no effect to maximum effect (from 0.31 to 1280 mg/kg per day in the experiment with the widest range, 2 mice per dose) administered every three hours (q3h) by subcutaneous injections of 200 μL. Two infected but untreated control mice were sacrificed right after inoculation (hour -2), at the onset (h0), and at the end of therapy (h24), when all other (treated) mice were euthanized and their thighs dissected under aseptic technique, homogenized, serially diluted, plated by duplicate on solid medium, and aerobically incubated at 37°C for 18 h. Data were registered as log_10_ CFU/g and the limit of detection was 2.0 log_10_ CFU/g; each thigh in this model weighs 1 g, therefore any thigh with zero colonies was entered in the database as 100 CFU/g. To determine net antibacterial effect, the number of CFU remaining in the thighs after 24 h of treatment was subtracted from the number of CFU that grew in the thighs of control mice during the same period.

#### Neutropenic mouse aerosolized pneumonia model

We standardized and optimized this model in order to evaluate in vivo the bactericidal efficacy of antibiotics in serious lung infections. We have found that the natural tendency of mice to group together during the aerosolization process increases the variance in the number of bacterial cells that reach the alveoli in each animal, preventing a lethal infection in the individuals that remained under all others. To deal with this problem, we first quantified the impact of the position and distribution of the animals within the aerosolization chamber during the inoculation process testing several options: (a) mice movement was restricted by placing groups of three within little mesh cages that prevented grouping and allowed an uniform distribution of all animals within the aerosolization chamber; (b) movement and grouping was unrestricted, allowing mice freedom to locate anywhere within the chamber; (c) same as the previous option, but preventing grouping by hand every time mice tried to regroup; and (d) half of the mice were caged and the other half were left free.

Option (a) above was selected for all experiments because it was the most effective to minimize variance of the inoculum size, bacterial growth in the lungs, and the time of death (100% by hour 45 post-exposure). The pneumonia model was then standardized within the following conditions: mice were immunosuppressed as in the thigh model and exposed during 45 min to an aerosol containing *K*. *pneumoniae* GRP-0107 (10^9^ log-phased CFU/mL); a control group of three was sacrificed 15 minutes later (i.e., one hour after starting aerosolization) finding in the lungs 5.04–6.47 log_10_ CFU/g (hour -14). Treatment (three animals per dose) started 14 h post-infection (h0) and ended 24 h later (h24), when animals were euthanized by cervical dislocation for aseptic lung processing and data registration as described above; untreated control groups of three mice each were also sacrificed at these time-points: h-14, h0 and h24. Two simultaneous experimental arms, one for the generic and one for the innovator of imipenem-cilastatin were treated along 24 h with doses ranging from no effect to maximum effect (10 to 1280 mg/kg per day) delivered q3h by subcutaneous injections of 200 μL.

#### Neutropenic mouse meningoencephalitis model

Using the same protocol for immunosuppression described for the thigh model, we inoculated bacteria directly in the brain by retro-orbital injection (10 μL) of a log-phased culture of *P*. *aeruginosa* GRP-0019, GRP-0049, GRP-0036, or ATCC 27853 (h-2). The size of the inoculum was different depending on the design of each experiment (see Results). Multiple doses ranging from 20–2560 mg/kg per day (ineffective to maximally effective) were administered q3h by subcutaneous injections of 200 μL. The experimental arms and the untreated controls (sterile saline) started therapy 2 h post-infection (h0) and ended 24 h later (h24), when mice were sacrificed by cervical dislocation and their brains dissected under aseptic technique and processed for colony counting and data registration.

### 4. Statistical analysis of in vivo data

A sigmoid dose-response model described by the Hill equation was used to analyze and determine in vivo efficacy by nonlinear regression (NLR):

$$E=\left(E_{max}*D^{N}\right)/{(ED}_{50}^{N}+D^{N}),$$

where *E* is the net antibacterial effect after 24 h of treatment (in log_10_ CFU/g), *E*_*max*_ is the maximum antibacterial effect (in log_10_ CFU/g), *D* is the imipenem-cilastatin dose (in mg/kg per day), *ED*_50_ is the effective dose needed to reach 50% of the *E*_*max*_ (in mg/kg per day), and *N* is the Hill’s slope. The primary pharmacodynamic parameters (PDP) obtained from the NLR of generic and innovator products were compared by the overall test for coincidence of the NLR, a specialized statistical technique for CFA (Prism 5.0) [22, 27]. Accepting a 5% chance for a type I error, the treatment of at least 10 animals per product to compare one generic with the innovator imipenem confers 99% power to reject the null hypothesis if the magnitude of the difference in antibacterial efficacy is ≥1.0 log_10_ CFU/g and the standard deviation (SD) of the residuals is <0.5 log_10_ CFU/g. Such difference between generic and the innovator represents a net bactericidal effect greater than 100,000 bacterial cells per gram of tissue, a threshold value several orders of magnitude greater than what would be considered important in clinical medicine.

Instead of the sigmoid Hill’s PD pattern expected in the animal model, the generic product displayed a paradoxical U-shaped dose-effect curve against *K*. *pneumoniae* GRP-0107 in the lung model. The best fit for such PD pattern is the Gaussian model, as described by Christopoulos for compounds with simultaneous agonistic and antagonistic actions [28], but if generic and innovator fit different PD models they are, by definition, therapeutically nonequivalent. To establish which model more appropriately described the dose-effect relationship of each imipenem product, we ran their respective NLR under both models, and then computed the probability of each model being properly fit using corrected Akaike’s Information Criteria (Prism 5.0).

## Results

### Susceptibility testing of imipenem products

S2 Table includes the geometric means and ranges (mg/L) for MIC, MBC and MBC/MIC ratio of generic and innovator against *S*. *aureus* GRP-0057, *K*. *pneumonia* GRP-0107, and *P*. *aeruginosa* strains ATTC 27853, GRP-0019, GRP-0049 and GRP-0036. The corresponding values of the quality control strains *S*. *aureus* ATCC 29213, *K*. *pneumoniae* ATCC 43816 and *Pseudomonas aeruginosa* ATCC 27853 stayed always within CLSI ranges. There were no differences between products.

### Microbiological assay

**Fig 1:**
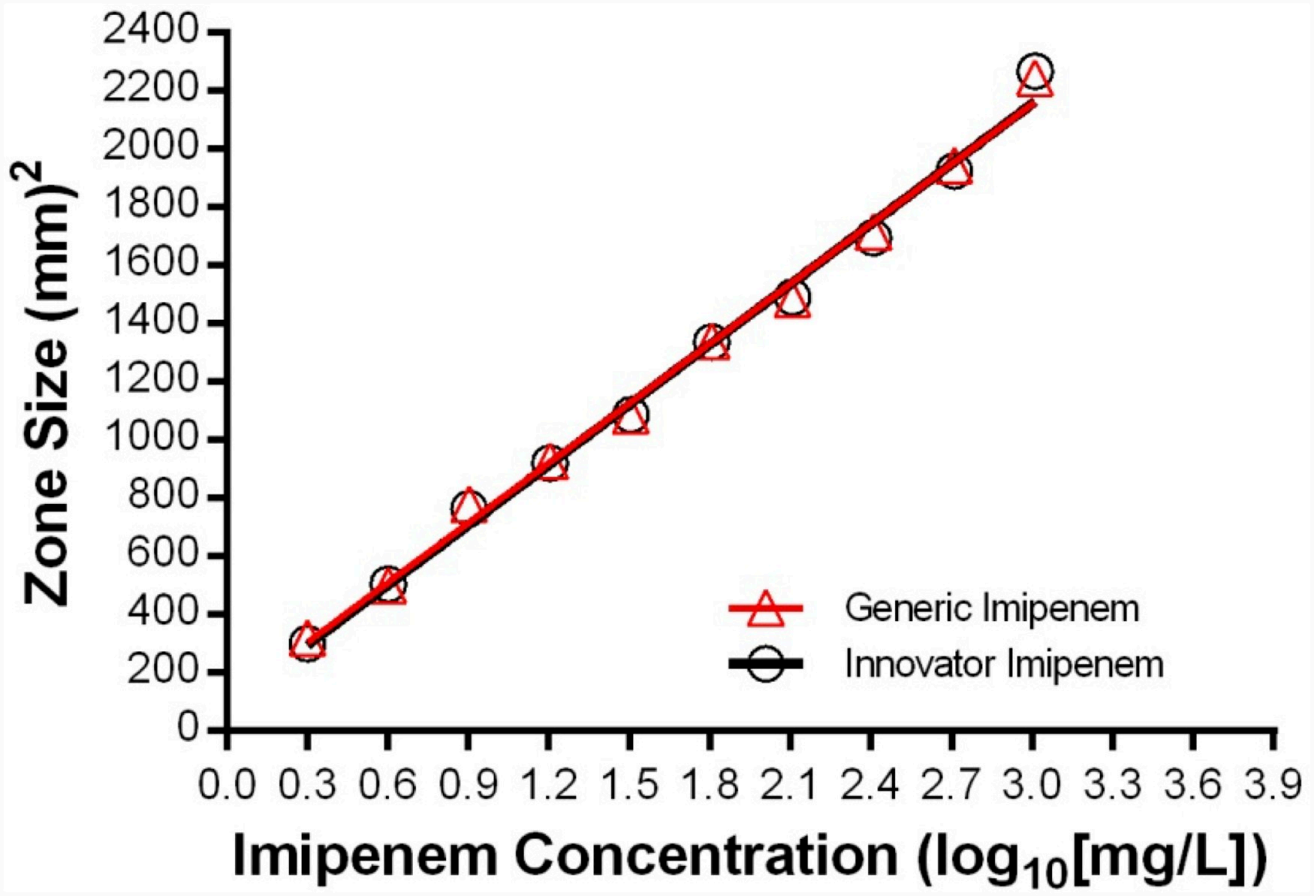
Pharmaceutical equivalence. Determination of concentration and potency of the active pharmaceutical ingredient of a generic product and the innovator of imipenem-cilastatin by microbiological assay. There are 12 data-points per drug but the size of the error bar is smaller than each symbol (P = 0.728 by CFA).

Fig 1 shows the standard curves generated by linear regression of generic and innovator demonstrating pharmaceutical equivalence, i.e., there was no difference in concentration (P_intercepts_ = 0.61) or potency (P_slopes_ = 0.62) of the API (imipenem). The nonlinear regression analysis demonstrated that all data in the graph belonged to the same population, therefore, it was fitted best by a single curve (P = 0.728; AdjR^2^ = 0.9807). However, the United States Pharmacopeia [29] requires for pharmaceutical equivalence of imipenem-cilastatin demonstration of no less than 90% and no more than 115% of the labeled amounts of both, imipenem (C_12_H_17_N_3_O_4_S·H_2_O; molecular weight 317.47) and cilastatin sodium (C_16_H_25_N_2_O_5_S Na; MW 380.43). Since the microbiological assay detects only antimicrobial compounds, it is necessary to use a different method to determine the amount of cilastatin before concluding that these products are pharmaceutical equivalents (see below).

### Imipenem standard curves by HPLC-UV

The chromatographic conditions were adjusted in order to provide a satisfactory performance of the assay. The calibration curves were constructed by plotting concentration versus analyte peak area and demonstrated good linearity in the 4–1000 μg/mL range. The accuracy of the method was determined and the mean recovery was found to be 90–100% indicating an agreement between the true value and the value found. The HPLC-UV method was suitably linear with a strong correlation between instrument response (peak area) and imipenem concentration (R ≥ 0.990). The detection and quantification limits went from 1 to 5,000 mg/L, respectively. The intra-day relative standard deviation was lower than 10%. Determination of API concentration (intercept) and potency (slope) in the reference, innovator and generic product did not show differences (P_intercepts_ = 0.854, P_slopes_ = 0.478) demonstrating, as did the bioassay, that the imipenem component of the generic product is pharmaceutically equivalent to the innovator under current regulations.

### Cilastatin quantification assay by HPLC-UV

**Fig 2:**
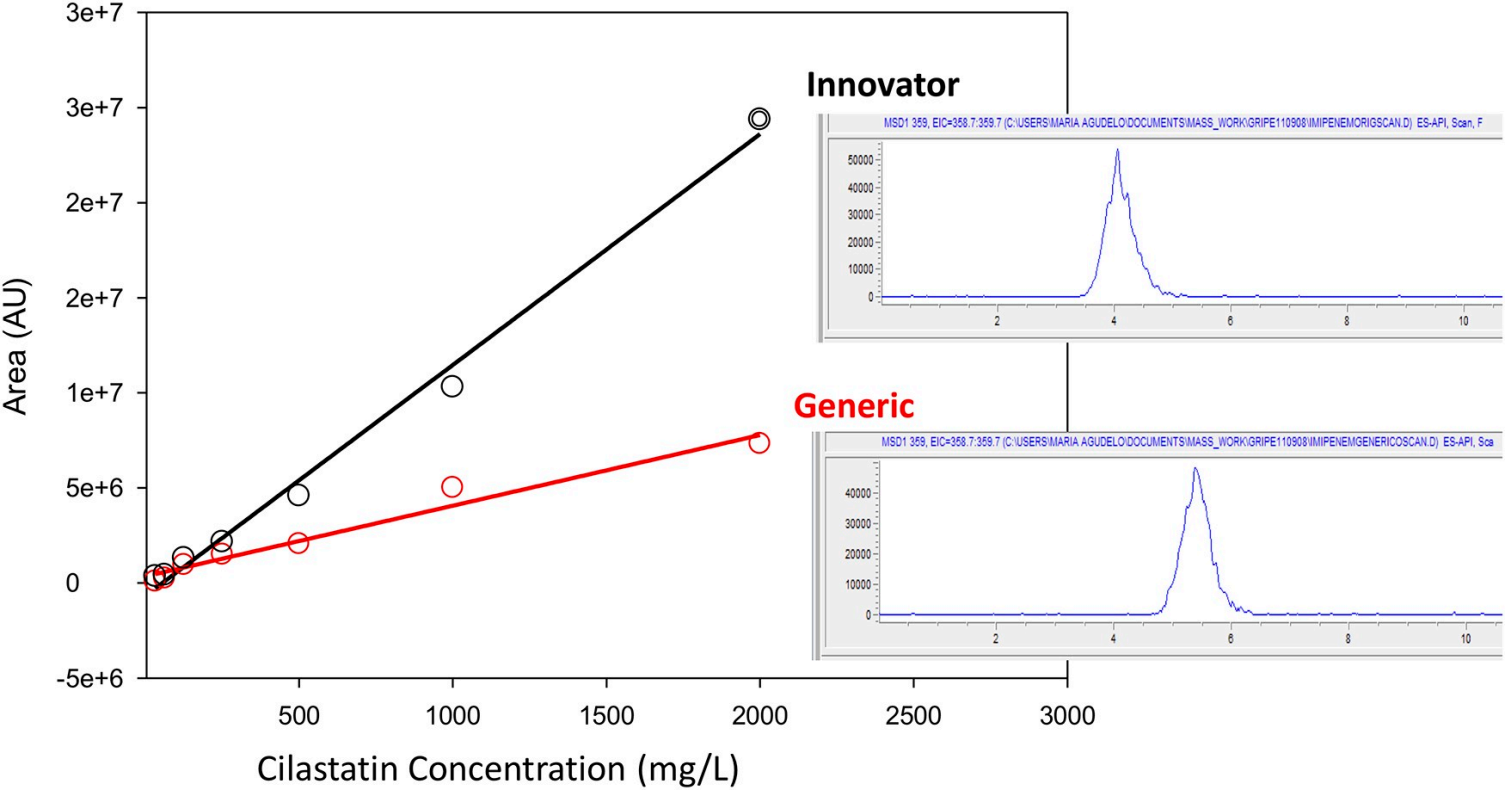
Cilastatin concentration. Quantitative assay by HPLC-UV for cilastatin concentration in a generic product and the innovator of imipenem; based on the chromatograms’ AUC for each product (right hand insets), the generic had a 30% lower concentration of the inhibitor with respect to the innovator.

The quantification of the pharmaceutical form was run simultaneously with the quantification of the API (this product needs 220 DO) and the generic product demonstrated 30% less concentration of cilastatin, implying an imipenem-to-cilastatin ratio of 1:0.7 instead of the innovator’s 1:1. Although we demonstrated that the concentration of the imipenem component was the same in both products, the generic did fail pharmaceutical equivalence under current regulations by having significantly lower concentration of the cilastatin component (Fig 2). In spite of this flagrant violation, none of the DRA of the different countries where this generic product was commercialized detected the problem.

### Imipenem stability assays

**Fig 3:**
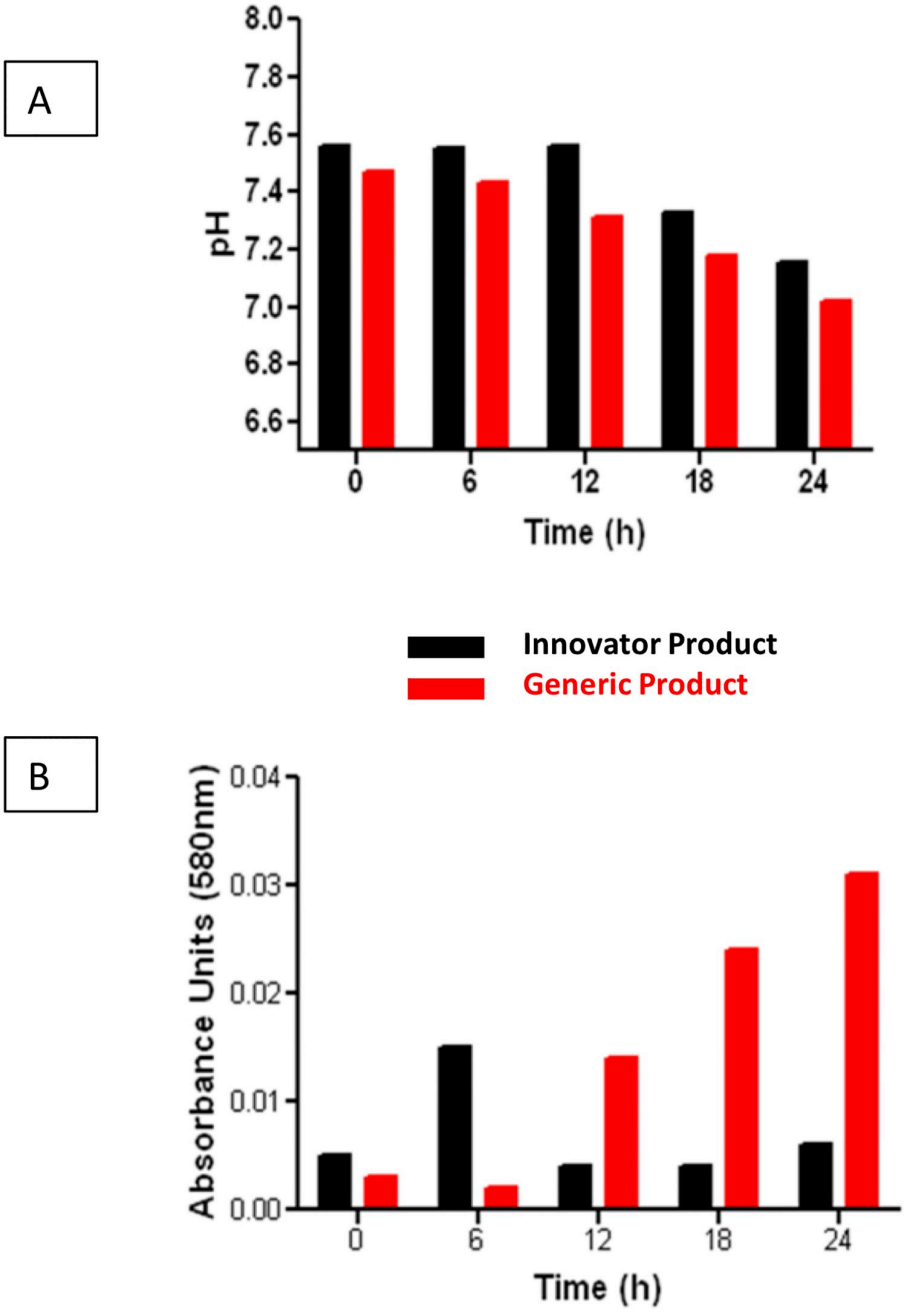
Chemical and physical stability. Comparison of pH (A) and colorimetric changes (B) at room temperature (25°C) of a generic product and the innovator of imipenem-cilastatin during the first 24 hours after powder reconstitution.

**Fig 4:**
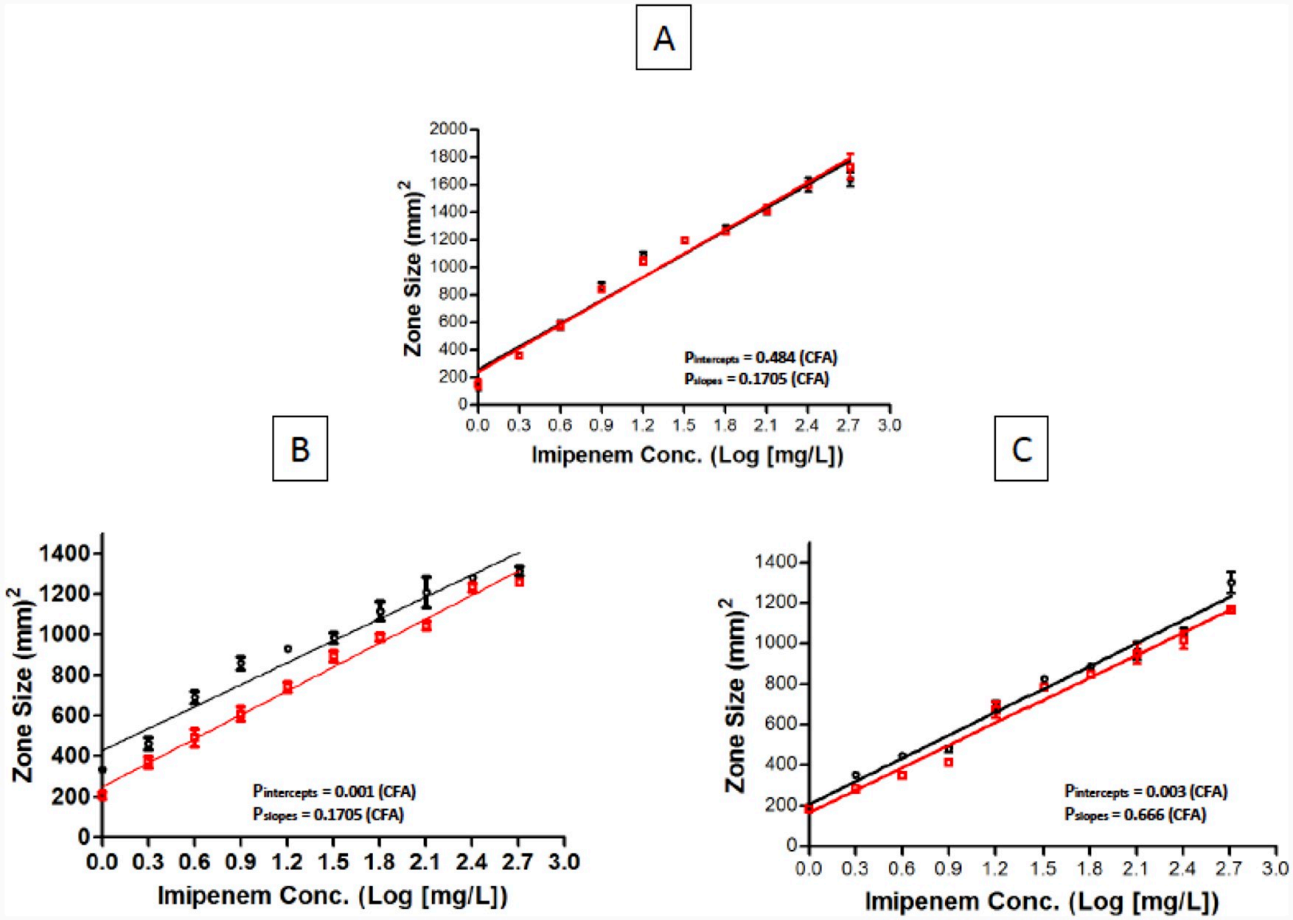
Concentration and potency. Comparison of a generic product and the innovator of imipenem-cilastatin immediately (A) and 24 hours after powder reconstitution standing at 4°C (B) and 25°C (C). There was no difference immediately after reconstitution in concentration or potency between innovator and generic. However, there were significant differences in potency after both products spent 24 hours at 4°C or 25°C because the generic was less stable than the innovator.

The degradation rate constant was temperature-dependent and confirmed an apparent first-order process; the most rapid degradation occurred at 37°C followed by 25°C. The generic product’s pH was lower (Fig 3A) and it exhibited a markedly higher absorbance along time (Fig 3B). In a similar fashion, both products started with the same potency and concentration of the API immediately after powder reconstitution (Fig 4A), but the generic suffered a significant decrease in concentration after 24 h of storage under refrigeration (Fig 4B) or at room temperature (Fig 4C). At 4°C, the innovator of imipenem is stable during 24 hours (and even 48 hours), but not the generic product; it caused a wide difference in potency evident in Fig 4B. However, both products are hydrolyzed at room (25°C) and physiological temperature (37°C), and the process becomes evident as early as hour 2. At 25°C, the generic suffers significantly greater degradation during 24 hours than the innovator, but at 37°C both products are hydrolyzed to the same extent. That explains why the curves of innovator and generic come closer as temperature increases (Fig 4C). Both products were equally hydrolyzed after 24 h at 37°C (not shown).

### Single-dose serum pharmacokinetics in neutropenic mice infected in the thighs with *P*. *aeruginosa* GRP-0019 (bioequivalence)

The two-compartment model with linear elimination and first-order absorption described better the pharmacokinetics of 3 doses (10, 20 y 40 mg/kg) with both imipenem products. Population parameter values are summarized in S3 Table and show that the imipenem component of the generic product was bioequivalent with respect to the innovator. The comparison of the geometric mean AUC generic/innovator ratio was 95% (90% CI 95–104), well within the 80% to 125% range accepted by DRA everywhere.

To determine if there were differences in the degree of precipitation between generic and innovator, we studied the PK of a fourth dose (80 mg/kg) that reach the solubility limit of imipenem-cilastatin (10 mg/mL) [30]. In fact, both products did show precipitation at this concentration, but the peak of the generic was one third lower than that of the innovator, suggesting that precipitation was greater in the generic formulation. The data obtained from this dose were not used to compute the population PK analysis.

### In vivo efficacy of the generic product and the innovator of imipenem-cilastatin against diverse pathogens in three animal models of infection (neutropenic mice)

S4 Table shows the number of bacterial cells and variance in untreated controls at the time of the inoculum (h-2 in the thigh and brain infection models, and h-14 in the pneumonia model) and when antibiotic treatment started (h0) and ended (h24), as well as the growth (G) of the pathogen in the animal tissues during the execution of the model. It demonstrates that animals develop active infections in the organs targeted by the respective model and explains their lethality, which is 100% at 48 h post-infection.

S5 Table lists the pathogen, model, PD parameter, nonlinear regression diagnostics for the dose-response curve of generic and innovator, as well as the result (P value) of the CFA.

### Neutropenic mouse thigh infection model with *S*. *aureus* GRP-0057 (WT)

**Fig 5:**
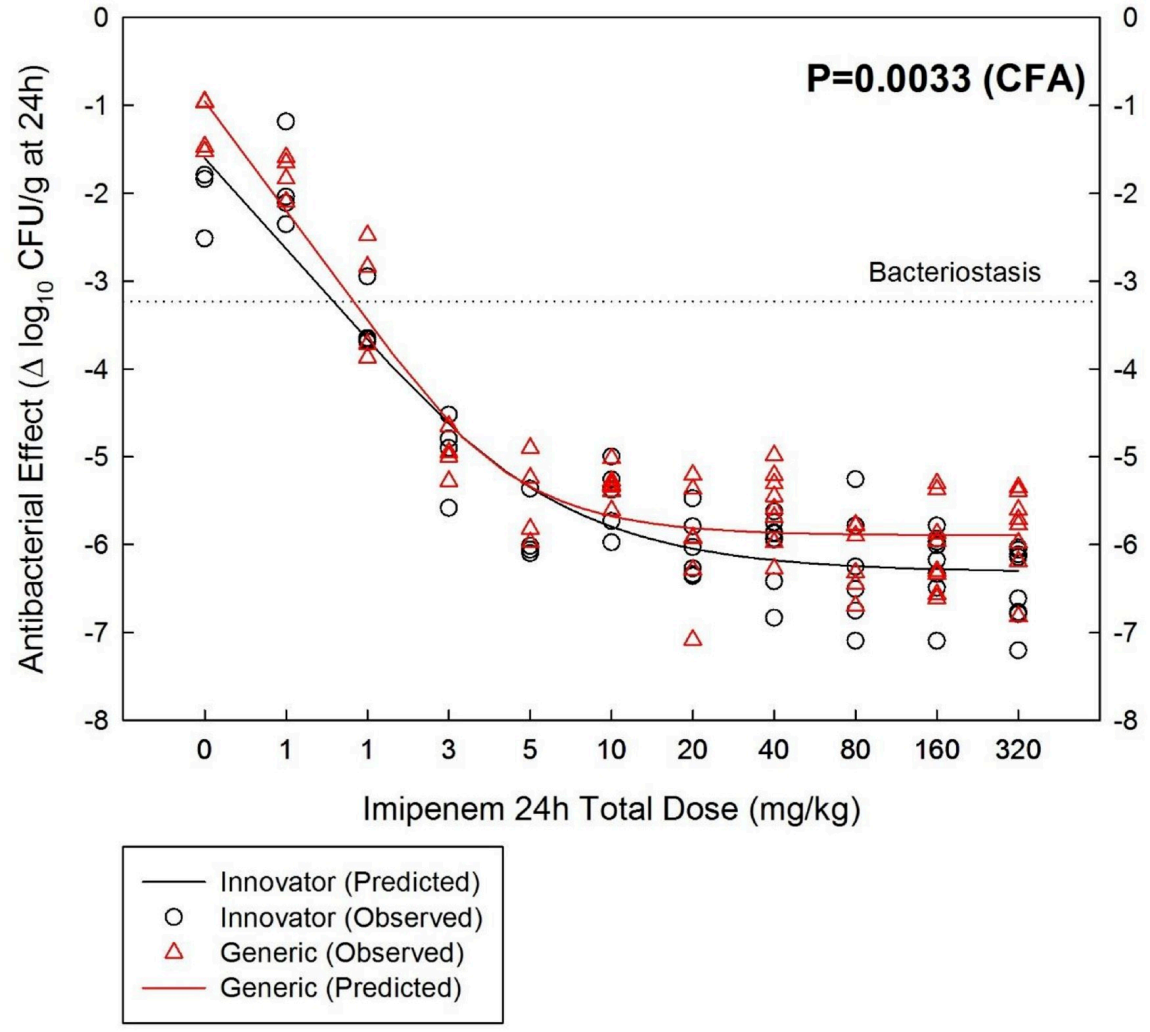
Pharmacodynamics of One Generic and the innovator of imipenem-cilastatin against the wild-type strain *S*. *aureus* GRP-0057 in the neutropenic mouse thigh infection model. The data shown in the graphs is pooled from 5 independent experiments comparing simultaneously both products, in which the generic was consistently less effective than the innovator.

The generic product was significantly less effective than the innovator against this strain and it was confirmed in several experiments (S5 Table). The data shown in the Fig 5 are pooled from 5 independent experiments in which the generic product was consistently less effective than the innovator (P<0.05 for each individual experiment). Their main differences were in the *E*_*max*_: 6.31 ± 0.11 vs. 5.90 ± 0.08 log_10_ CFU/g in 24h for innovator and generic, respectively (P = 0.0033 by CFA). A sixth experiment widening the dosing-range gave identical results (S5 Table).

### In vivo efficacy against *K*. *pneumoniae* GRP-0107 (WT) of generic and innovator in the neutropenic mouse aerosolized pneumonia model

**Fig 6:**
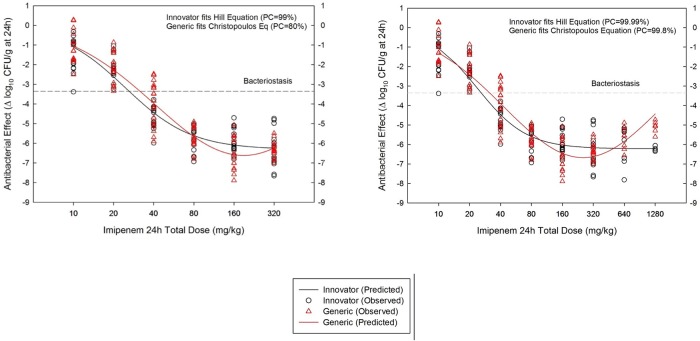
Pharmacodynamics (PD) of One Generic and the innovator of imipenem-cilastatin against the wild-type strain *K*. *pneumoniae* GRP-0107 in the neutropenic mouse lung infection model. Opposite to the innovator, the generic product fitted a Gaussian instead of the Hill equation, displaying a well characterized Eagle effect. By definition, a generic with a different PD behavior is pharmaceutically nonequivalent (left panel). Increasing the dose above the limit of solubility did not affect the innovator, but deepened the Eagle effect of the generic, demonstrating that precipitation was not the cause of this aberrant PD pattern (right panel).

This model represents a harder PD challenge for the antibiotic not only on account of the targeted organ, but also because *K*. *pneumoniae* GRP-0107 (MIC = 0.5 mg/L) is 33-times less susceptible to imipenem than *S*. *aureus* GRP-0057 (MIC = 0.015 mg/L). It explains the 30-fold larger amount of drug required to reach 50% of the *E*_*max*_, which corresponds to the primary PDP *ED*_*50*_, the mathematical expression of the potency of the antibiotic (S5 Table). In order to reach maximal efficacy, the lung model required much greater doses of imipenem that, without reaching the limit of solubility, revealed a conspicuous Eagle effect in the generic product described by the Christopoulos equation [28]. It consists in a paradoxical and progressive decline in efficacy in response to increments in the dose (Fig 6A). When this pharmacodynamic pattern was analyzed with corrected Akaike’s information criteria to determine if the Eagle effect was in fact describing the true behavior of the generic product, it was confirmed with an 80% probability of correctness, indicating that only the Christopoulos equation could fit its dose-response curve. The innovator’s pharmacodynamics, on the contrary, were described by Hill’s equation with 99% probability of correctness.

To rule out the possibility of imipenem precipitation, we used higher doses in the animal model (up to twice the limit of solubility, i.e., 160 mg/kg per dose, 20 mg/mL): the Eagle effect became more pronounced with the generic product, which fitted the Christopoulos equation with a 99.8% probability of correctness, while it was not seen with the innovator, which remained fitting the Hill equation with a 99.99% probability of correctness (Fig 6B). A generic product which PD pattern is not the same of the innovator is, by definition, therapeutically nonequivalent [3].

### In vivo efficacy against several strains of *P*. *aeruginosa* in the neutropenic mouse meningoencephalitis model

Elimination of *P*. *aeruginosa* infecting the central nervous system is particularly difficult considering the toughness of the pathogen and the blockade of imipenem and cilastatin by the blood-brain-barrier, which allows only 20% to 30% of each compound to reach the cerebrospinal fluid with inflamed meninges [31, 32]. This problem forced the use of very high doses, including 80 and 160 mg/kg per dose, which correspond to once (10 mg/mL) and twice (20 mg/mL) the limit of solubility for imipenem [30]. If, as required by DRA agencies worldwide and as inscribed in the label of both products, generic and innovator are identical chemical entities, overcoming the limit of solubility should not in itself affect their pharmacodynamic equivalence. In fact, the innovator has been used at higher doses (200 mg/kg) in mice to test for toxicity [33].

**Fig 7:**
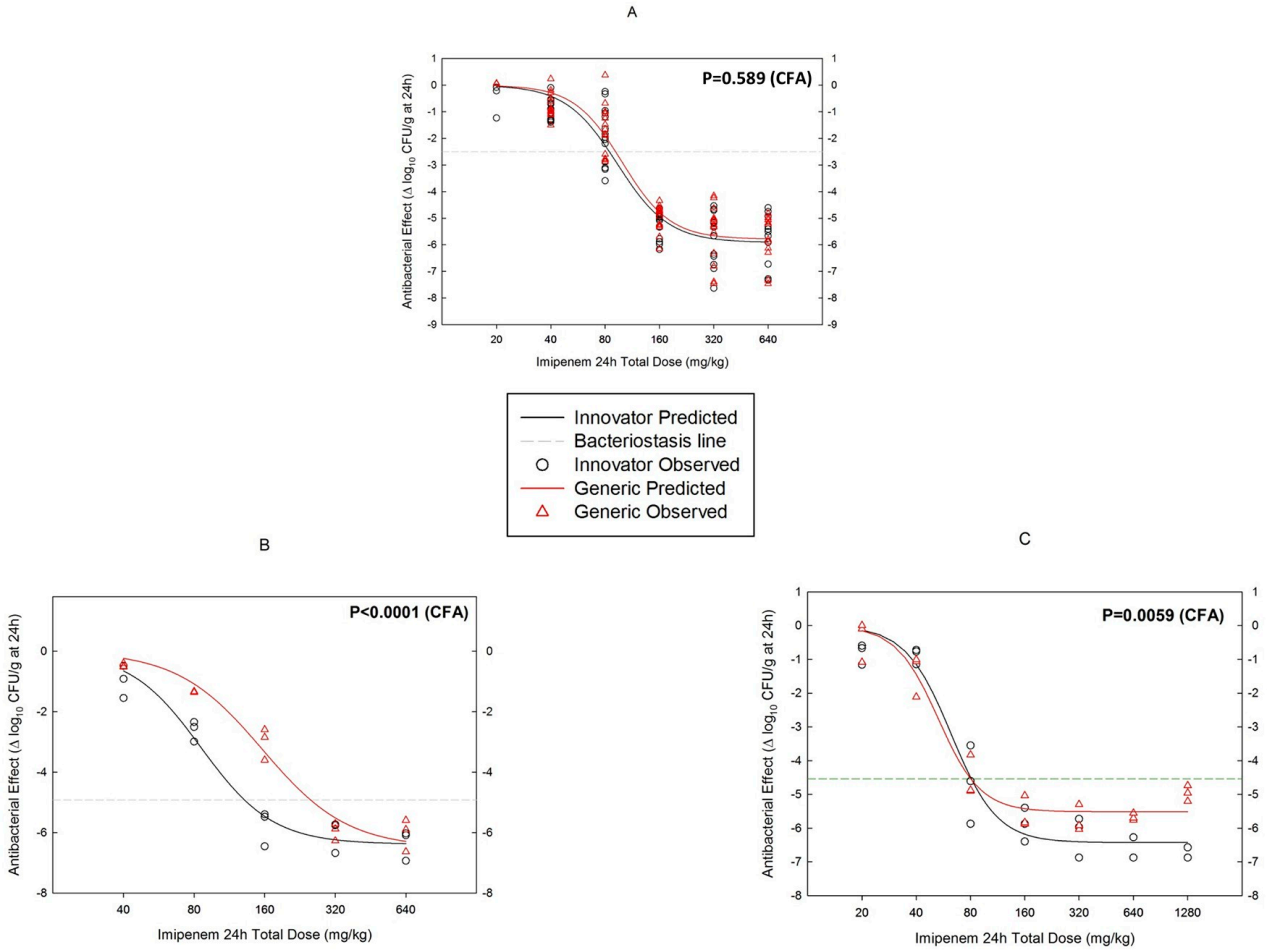
Pharmacodynamics (PD) of One Generic and the innovator of imipenem-cilastatin against several strains of *P*. *aeruginosa* in the neutropenic mouse brain infection model. There was no difference against *P*. *aeruginosa* GRP-0019, the strain with the lowest imipenem MIC (0.5 mg/L), as illustrated by the overlapping dose-effect curves (A). However, therapeutic nonequivalence was evident against the less susceptible strains *P*. *aeruginosa* ATCC 27853 (B) and *P*. *aeruginosa* GRP-0049 (C). As expected, the carbapenem-resistant *P*. *aeruginosa* GRP-0036 was untreatable in this model (not shown).

No difference was detected in the pharmacodynamic profiles of generic and innovator imipenem-cilastatin against the wild-type strain *P*. *aeruginosa* GRP-0019 (MIC = 0.5 mg/L), a finding that was confirmed in five independent experiments in which treatment started against inoculum sizes of 4, 5, or 6 log_10_ CFU/g of brain, the highest of which was untreatable (S4 and S5 Tables). Fig 7A shows the overlapping dose-response curves of generic and innovator against this strain after fitting the data from all five experiments (the high variance is explained by the widely different inocula used in each experiment).

The result with the other three strains of *P*. *aeruginosa* in the meningoencephalitis model confirmed the lack of therapeutic equivalence of the generic product (inoculum sizes are shown in S4 Table). Against *P*. *aeruginosa* ATCC 27853 (MIC = 1.0 mg/L), a highly significant difference was evident between generic and innovator because the former required twice the dose of the latter to reach bacteriostasis: 251.4 ± 22.1 vs. 131.2 ± 12.2 (P<0.0001; Fig 7B). Against *P*. *aeruginosa* GRP-0049 (MIC = 1.2 mg/L), the generic product was less effective instead of less potent than the innovator (Fig 7C): *E*_*max*_, 5.51 ± 0.153 vs. 6.43 ± 0.196 log_10_ CFU/g in 24 h, respectively (P = 0.0059), with the same bacteriostatic dose (80.4 and 80.7 mg/kg per day). The fully resistant strain *P*. *aeruginosa* GRP-0036 (MIC = 8 mg/L) was untreatable with imipenem at the highest doses (data not shown).

### LC/MS analysis of generic and innovator imipenem

**Fig 8:**
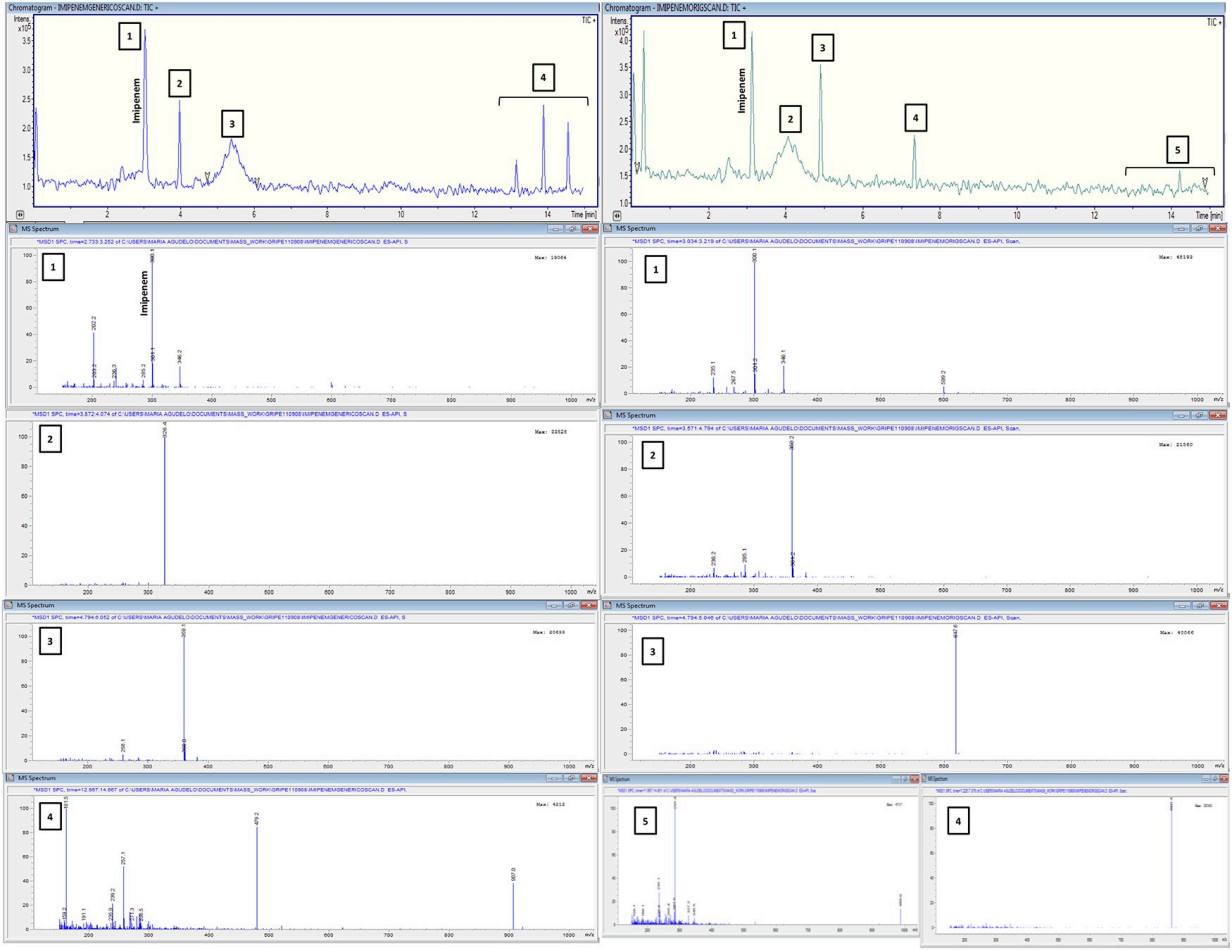
LC/MS SCAN mode (range, *m/z* 100–1000) of the pharmaceutical forms of the generic product and the innovator of imipenem-cilastatin (Fresh Samples). Left (generic) and right (innovator) panels show the spectrogram (up) and under it the centroids graphs describing the composition masses of each peak numbered. There were no differences in the analyte signal (peak 1 in both panels), i.e., the active pharmaceutical ingredient (m/z 300) is present in both products at the same concentration. However, the generic product exhibited different structural information in the full scan analysis, represented in peaks that expressed masses (peaks 2, 3, 4) absent in the innovator (which peaks 2, 3, 4, and 5 correspond to other masses). The peaks named 4 in the generic (left panel) and 5 in the innovator (right panel) have different abundance for the same concentration (250 mg/L).

The scan analysis was done in a range of m/z 150–1000 during 15 min. For both products in solution, the MS spectrum of protonated sample (m/z 300) undergoes common fragmentation routes described in previous studies [34]. The analysis identified 3 main product ions at m/z 257, 235, and 285, generated during the dissociation of the parental compound or its adducts (Fig 8). This fragmentation is attributed to the facile loss of a sulfur atom or to neutral losses. Interestingly, the generic product showed a fragmentation channel at m/z 326 much more frequently than the innovator. It could be formed after the specific loss of a nitrogen atom in a hydrogen cyanide reaction (HCN), with a subsequent gain of a sodium atom by means of a CHNa reaction provided by the sodium carbonate (Na_2_CO_3_) present as an excipient in the pharmaceutical form. Alternatively, the presence of this product could rest in a greater capacity of the generic to conform dimers (m/z 669) that, as detected in the deconvolution analysis, lost several structures (181-235-344- or 344-NH_4_) during the fragmentation process. This finding is related with the detection of a major abundance of the dimeric structures in the total mass spectrum analysis (Fig 8).

## Discussion

The data demonstrate that this generic of imipenem-cilastatin and the innovator have the same concentration of the API (i.e., imipenem), but the generic product failed pharmaceutical equivalence due to a substandard amount of the DHP-I inhibitor cilastatin (30% less than announced). However, the generic fulfilled DRA criteria for PK equivalence because its imipenem component reach the same concentration of the innovator in blood, which suggests that imipenem was protected from DHP-1 hydrolysis with only 70% of the required amount of cilastatin [4]. In addition to failing pharmaceutical equivalence, the generic also failed therapeutic equivalence in most (but not all) of our animal models, indicating that there must be other reasons to explain nonequivalence of this product.

Although it was licensed as pharmaceutical equivalent and “bioequivalent” by DRA in Colombia (and in every other country in which it was commercialized), the generic product of imipenem-cilastatin had gross chemical differences with its comparator: (1) it contains 30% less cilastatin than required; (2) the API of the generic is highly unstable once in solution; (3) it has lower pH after dissolution and faster acidification rate than the innovator; and (4) the degradation process of the generic ends up in four different products not present in the innovator. The four unexpected degradation products may result from transformation of impurities or from molecular reorganization due to different thermodynamics. Together, these faults help to understand the different pharmacodynamics of this generic of imipenem-cilastatin.

The only result suggesting imipenem hydrolysis in vivo (attributable to the lower amount of cilastatin) was seen in the brain model with *P*. *aeruginosa* ATCC 27853, where generic imipenem was twice less potent than the innovator (Fig 7B). There are only two potential sources of imipenem hydrolysis, the murine DHP-I (3.3-fold more active against imipenem than its human homologue) and bacterial beta-lactamases. It could not be explained by murine DHP-I hydrolysis because (1) the production of the enzyme in the brain is extremely low [35], (2) the problems exhibited by this generic against the other three strains of *P*. *aeruginosa* in the brain model were not related to potency, and (3) there was no evidence of hydrolysis in the lung model, an organ with 3,000-fold more DHP-I than the brain [36, 37]. This leaves bacterial beta-lactamases as the only explanation. Cilastatin is included in the formulation of imipenem as a competitive inhibitor of DHP-I, a zinc metalloenzyme of the membrane dipeptidase family (M19), which encompasses enzymes from diverse kingdoms including animals and bacteria [38]. M19 enzymes are versatile in their substrate specificity and hydrolyze dipeptides, dehydropeptides and beta-lactams of the *trans*-conformation (like imipenem), but cilastatin is a very inefficient inhibitor of bacterial dipeptidases [39]. Although *P*. *aeruginosa* ATCC 27853 is a wild-type strain, all members of this species produce a membrane dipeptidase called CdhR (a transcriptional regulator of carnitine catabolic genes) that, belonging to the M19 family and being homologue of the mammalian DHP-I, could have hydrolytic activity against carbapenems [40–42].

There were other two forms of nonequivalence of generic imipenem in this study, one in the thigh model against the wild type *S*. *aureus* strain GRP-0057, and the other in the brain model against the multidrug resistant strain *P*. *aeruginosa* GRP-0049; both can be explained by chemical inequalities. The instability of the generic product results first and foremost in the opening of the beta lactam ring [43], which renders imipenoic acid, a molecule that is virtually identical to imipenem but devoid of antibacterial effect. It can compete for the molecular target with intact imipenem, or even displace intact imipenem from its target (3). The net result from this kind of antagonism is the Eagle effect, a paradoxical pharmacodynamic pattern of lower efficacy with higher doses [42]. But the generic product was not only unstable, it had several impurities and degradation products very similar to the API that could enhance the Eagle effect. Special conditions of the microorganism and the organ targeted in each model would define what mechanism was prevalent, but probably all of them work together with different degrees of interference.

In conclusion, we provide experimental demonstration of a generic product of imipenem-cilastatin failing therapeutic equivalence through several mechanisms. Strictly speaking, this generic is not a pharmaceutical equivalent of the innovator because it contains 30% less cilastatin, but it did not prevent its DRA licensing and prescription in many countries. This gross fault went undetected for years because it did not affect equivalence in terms of MIC, MBC, in vitro concentration and potency of the API, or serum pharmacokinetics, and it was uncovered only by using animal models of infection. Surprisingly, the lower amount of cilastatin was not the only contributor to therapeutic nonequivalence. A more acidic formulation on top of a faster acidification favored the instability of the API (imipenem) after dissolution, and four degradation products with high similarity to the API antagonized its efficacy in vivo causing a lower *E*_*max*_ or, depending on the experimental conditions, the classical agonist-antagonist interaction known as the Eagle effect. Once again, in vivo data show that therapeutic equivalence requires more than just “bioequivalence” of the API, and some evidence is already pointing to drugs other than antimicrobials [44].

## Supporting information

S1 Table Characteristics of the pharmaceutical products of imipenem-cilastatin included in the study. (DOCX)

S2 Table Determination of minimal inhibitory (MIC) and bactericidal (MBC) concentrations of one generic and the innovator product of imipenem-cilastatin against the diverse bacterial strains employed in the study. (DOCX)

S3 Table Population pharmacokinetics. Comparison of one generic product of imipenem and the innovator (bioequivalence) in neutropenic mice infected in the thighs with *P*. *aeruginosa* GRP-0019.
(DOCX)

S4 Table Basic information for the animal models employed in the study. Bacterial load of untreated controls at the time of the inoculum (h-2 or h-14) and when therapy with imipenem-cilastatin started (h0) and ended (h24), as well as bacterial growth (G) along the 24-hour treatment period, dose-range and dosing intervals designed for each infection model.
(DOCX)

S5 Table Pharmacodynamic parameters, regression diagnostics and statistical analysis. Primary pharmacodynamic parameters (*E*_*max*_, *ED*_*50*_, *N*) obtained by nonlinear regression, regression diagnostics, and statistical comparison by curve fitting analysis of the dose-response data from a generic product and the innovator of imipenem-cilastatin in diverse animal models of infection.
(DOCX)

## References

[1] Zuluaga AF, Agudelo M, Cardeño JJ, Rodriguez CA, Vesga O. Determination of therapeutic equivalence of generic products of gentamicin in the neutropenic mouse thigh infection model. PLoS One. 2010; 5:e10744 10.1371/journal.pone.0010744 20505762 PMC2873963

[2] Rodriguez CA, Agudelo M, Zuluaga AF, Vesga O. In vitro and in vivo comparison of the anti-staphylococcal efficacy of generic products and the innovator of oxacillin. BMC Infect Dis. 2010; 10:153 10.1186/1471-2334-10-153 20525378 PMC2897798

[3] Vesga O, Agudelo M, Salazar BE, Rodriguez CA, Zuluaga AF. Generic vancomycin products fail in vivo despite being pharmaceutical equivalents of the innovator. Antimicrob Agents Chemother. 2010; 54:3271–9. 10.1128/AAC.01044-09 20547818 PMC2916296

[4] Agudelo M, Rodriguez CA, Pelaez CA, Vesga O. Even apparently insignificant chemical deviations among bioequivalent generic antibiotics can lead to therapeutic nonequivalence: the case of meropenem. Antimicrob Agents Chemother. 2014; 58:1005–18. 10.1128/AAC.00350-13 24277034 PMC3910812

[5] Zuluaga AF, Rodriguez CA, Agudelo M, Vesga O. Pharmacodynamics of nine generic products of amikacin compared with the innovator in the neutropenic mouse thigh infection model. BMC Res Notes. 2015; 8:546 10.1186/s13104-015-1507-z 26445936 PMC4596513

[6] Rodriguez CA, Agudelo M, Zuluaga AF, Vesga O. In vivo pharmacodynamics of piperacillin/tazobactam: implications for antimicrobial efficacy and resistance suppression with innovator and generic products. Int J Antimicrob Agents. 2017 2;49(2):189–197. 10.1016/j.ijantimicag.2016.10.011 27988068

[7] Agudelo M, Vesga O. Therapeutic equivalence requires pharmaceutical, pharmacokinetic, and pharmacodynamic identities: true bioequivalence of a generic product of intravenous metronidazole. Antimicrob Agents Chemother. 2012; 56:2659–65. 10.1128/AAC.06012-11 22330928 PMC3346647

[8] Rodriguez CA, Agudelo M, Zuluaga AF, Vesga O. Impact on resistance of the use of therapeutically equivalent generics: the case of ciprofloxacin. Antimicrob Agents Chemother. 2015; 59:53–8. 10.1128/AAC.03633-14 25313208 PMC4291395

[9] Gonzalez JM, Rodriguez CA, Zuluaga AF, Agudelo M, Vesga O. Demonstration of therapeutic equivalence of fluconazole generic products in the neutropenic mouse model of disseminated candidiasis. PLoS One. 2015; 10(11):e0141872 10.1371/journal.pone.0141872 26536105 PMC4633286

[10] Agudelo M, Rodriguez CA, Zuluaga AF, Vesga O. Relevance of various animal models of human infections to establish therapeutic equivalence of a generic product of piperacillin/tazobactam. Int J Antimicrob Agents. 2015; 45:161–7. 10.1016/j.ijantimicag.2014.10.014 25481459

[11] Fujimura S, Watanabe A. Generic antibiotics in Japan. J Infect Chemother. 2012; 18(4):421–7. 10.1007/s10156-012-0437-0 22684334

[12] Generic Pharmaceutical Association. Generic drug savings in the U.S. Sixth Annual Edition: 2014. Accessed April 19, 2016, http://www.gphaonline.org/media/cms/GPhA_Savings_Report.9.10.14_FINAL.pdf

[13] Mastoraki E, Michalopoulos A, Kriaras I, Mouchtouri E, Falagas ME, Karatza D et al Incidence of postoperative infections in patients undergoing coronary artery bypass grafting surgery receiving antimicrobial prophylaxis with original and generic cefuroxime. J Infect. 2008; 56:35–9. 10.1016/j.jinf.2007.09.011 17983660

[14] Rodriguez CA, Agudelo M, Cataño JC, Zuluaga AF, Vesga O. Potential therapeutic failure of generic vancomycin in a liver transplant patient with MRSA peritonitis and bacteremia. J Infect. 2009; 59:277–80. 10.1016/j.jinf.2009.08.005 19698745

[15] Rodriguez CA, Agudelo M, Zuluaga AF, Vesga O. Generic vancomycin enriches resistant subpopulations of Staphylococcus aureus after exposure in a neutropenic mouse thigh infection model. Antimicrob Agents Chemother. 2012; 56:243–7. 10.1128/AAC.05129-11 22064531 PMC3256022

[16] Rodriguez CA, Agudelo M, Aguilar YA, Zuluaga AF, Vesga O. Impact on Bacterial Resistance of Therapeutically Nonequivalent Generics: The Case of Piperacillin-Tazobactam. PLoS One. 2016 5 18;11(5):e0155806 10.1371/journal.pone.0155806 27191163 PMC4871539

[17] Alliance for Human Research Protection. Former FDA Commissioner says FDA lost public trust: 17 Oct 2005. Accessed April 19, 2016, http://ahrp.org/former-fda-commissioner-says-fda-lost-public-trust/].

[18] Papp-Wallace KM, Endimiani A, Taracila MA, Bonomo RA. Carbapenems: past, present, and future. Antimicrob Agents Chemother. 2011; 55:4943–60. 10.1128/AAC.00296-11 21859938 PMC3195018

[19] Clinical and Laboratory Standards Institute. 2012. Performance standards for antimicrobial susceptibility testing, approved standard M100-S22. Clinical and Laboratory Standards Institute, Wayne, PA.

[20] Zuluaga AF, Agudelo M, Rodriguez CA, Vesga O. Application of microbiological assay to determine pharmaceutical equivalence of generic intravenous antibiotics. BMC Clin Pharmacol. 2009; 9:1 10.1186/1472-6904-9-1 19149891 PMC2640365

[21] Bennett JV, Brodie JL, Benner EJ, Kirby WM. Simplified, accurate method for antibiotic assay of clinical specimens. Appl Microbiol. 1966; 14:170–7. 4959982 10.1128/am.14.2.170-177.1966 PMC546645

[22] Glantz SA. 2002 How to test for trends, p. 230–297. In Glantz S. A. (ed.), Primer of biostatistics, 5th ed McGraw-Hill Companies, Inc., New York, NY.

[23] Myers CM, Blumer JL. Determination of imipenem and cilastatin in serum by high-pressure liquid chromatography. Antimicrob Agents Chemother. 1984; 26:78–81. 6591852 10.1128/aac.26.1.78 PMC179921

[24] Louie A, Bied A, Fregeau C et al Impact of different carbapenems and regimens of administration on resistance emergence for three isogenic Pseudomonas aeruginosa strains with differing mechanisms of resistance. Antimicrob Agents Chemother. 2010; 54:2638–45. 10.1128/AAC.01721-09 20308371 PMC2876389

[25] Neely M, van Guilder M, Yamada W, Schumitzky A, Jelliffe R. Accurate detection of outliers and subpopulations with Pmetrics, a nonparametric pharmacometric modeling and simulation package for R. Ther Drug Monit 2012; 34:467–76. 10.1097/FTD.0b013e31825c4ba6 22722776 PMC3394880

[26] Zuluaga AF, Salazar BE, Rodriguez CA, Zapata AX, Agudelo M, Vesga O. Neutropenia induced in outbred mice by a simplified low-dose cyclophosphamide regimen: characterization and applicability to diverse experimental models of infectious diseases. BMC Infect Dis. 2006; 6:55 10.1186/1471-2334-6-55 16545113 PMC1434751

[27] Motulsky H, Christopoulos A. Fitting models to biological data using linear and nonlinear regression. New York (NY): Oxford University Press; 2004.

[28] Christopoulos A, Grant MK, Ayoubzadeh N, Kim ON, Sauerberg P, Jeppesen L et al Synthesis and pharmacological evaluation of dimeric muscarinic acetylcholine receptor agonists. J Pharmacol Exp Ther. 2001; 298:1260–8. 11504829

[29] Unites States Pharmacopoeia (USP)—National Formulary [USP 29—NF 24]. Volume 27(1). Rockville, MD: United States Pharmacopeial Convention, Inc; 2005. [MDANT05] Monograph Development—Antibiotics; p. 1110.

[30] National Center for Biotechnology Information. PubChem Compound Database; CID = 104838, https://pubchem.ncbi.nlm.nih.gov/compound/104838 (accessed May 5, 2017).

[31] Patamasucon P, McCracken GH Jr.. Pharmacokinetics and bacteriological efficacy of N-formimidoyl thienamycin in experimental Escherichia coli meningitis. Antimicrob Agents Chemother. 1982; 21:390–2. 7049073 10.1128/aac.21.3.390 PMC181901

[32] Jacobs RF, Kearns GL, Brown AL, Longee DC. Cerebrospinal fluid penetration of imipenem and cilastatin (primaxin) in children with central nervous system infections. Antimicrob Agents Chemother. 1986; 29:670–4. 3458427 10.1128/aac.29.4.670 PMC180464

[33] Merck. Primaxin, product monograph (imipenem and cilatatin sodium for injection, USP), I.V. infusion, antibiotic. Kirkland (Quebec). Merck Canada Inc. August 25, 2015, http://www.merck.ca/assets/en/pdf/products/PRIMAXIN-PM_E.pdf (accessed May 5, 2017).

[34] Xia M, Hang TJ, Zhang F, Li XM, Xu XY. The stability of biapenem and structural identification of impurities in aqueous solution. J Pharm Biomed Anal. 2009; 49:937–44. 10.1016/j.jpba.2009.02.002 19278804

[35] Kera Y, Liu Z, Matsumoto T, Sorimachi Y, Nagasaki H, Yamada RH. Rat and human membrane dipeptidase: tissue distribution and developmental changes. Comp Biochem Physiol B Biochem Mol Biol. 1999; 123:53–8. 10425712 10.1016/s0305-0491(99)00039-5

[36] Fukasawa M, Sumita Y, Harabe ET, Tanio T, Nouda H, Kohzuki T et al Stability of meropenem and effect of 1 beta-methyl substitution on its stability in the presence of renal dehydropeptidase I. Antimicrob Agents Chemother. 1992; 36:1577–9. 1510457 10.1128/aac.36.7.1577 PMC191626

[37] Rawlings ND, Barrett AJ, Bateman A. (2012) MEROPS: the database of proteolytic enzymes, their substrates and inhibitors. Nucleic Acids Res 40, D343–D350. Specific information on family M19 https://merops.sanger.ac.uk/cgi-bin/famsum?family=m19 (accessed April 19, 2016). 10.1093/nar/gkr987 22086950 PMC3245014

[38] Keynan S, Hooper NM, Felici A, Amicosante G, Turner AJ. The renal membrane dipeptidase (dehydropeptidase I) inhibitor, cilastatin, inhibits the bacterial metallo-beta-lactamase enzyme CphA. Antimicrob Agents Chemother. 1995; 39:1629–31. 7492120 10.1128/aac.39.7.1629 PMC162797

[39] Wargo MJ, Hogan DA. Identification of genes required for Pseudomonas aeruginosa carnitine catabolism. Microbiology 2009; 155:2411–9. 10.1099/mic.0.028787-0 19406895 PMC2857723

[40] Cummings JA, Nguyen TT, Fedorov AA, Koln P, Xu C, Fedorov EV et al Structure, mechanism, and substrate profile for Sco3058: the closest bacterial homologue to human renal dipeptidase. Biochemistry. 2010; 49(3):611–22. 10.1021/bi901935y 20000809 PMC2808448

[41] Winsor GL, Lam DK, Fleming L et al Pseudomonas Genome Database: improved comparative analysis and population genomics capability for Pseudomonas genomes. Nucleic Acid Res. 2011; 39(Database issue):D596–600. Specific information on gene PA5389 http://www.pseudomonas.com/feature/show?id=113662 (accessed April 19, 2016). 10.1093/nar/gkq869 20929876 PMC3013766

[42] Eagle H, Musselman AD. The rate of bactericidal action of penicillin in vitro as a function of its concentration, and its paradoxically reduced activity at high concentrations against certain organisms. J Exp Med. 1948; 88:99–131. 18871882 10.1084/jem.88.1.99 PMC2135799

[43] Cielecka-Piontek J, Michalska K, Zalewski P, Jelińska A. Recent advances in stability studies of carbapenems. Current Pharmaceutical Analysis 2011; 7:213–227.

[44] Meredith PA. Potential concerns about generic substitution: bioequivalence versus therapeutic equivalence of different amlodipine salt forms. Curr Med Res Opin. 2009; 25:2179–89. 10.1185/03007990903116867 19601710

