# Supplementary material for: Nontherapeutic equivalence of a generic product of imipenem-cilastatin is caused more by chemical instability of the active pharmaceutical ingredient (imipenem) than by its substandard amount of cilastatin
Source: PLoS One. 2019 Feb 6;14(2):e0211096. doi: 10.1371/journal.pone.0211096 (PMC6364906; doi:10.1371/journal.pone.0211096)
Supplement: S1 Table — (DOCX) [file pone.0211096.s001.docx]

**S1 Table. Characteristics of the Pharmaceutical Products of Imipenem-Cilastatin Included in the Study.**

| **Imipenem Product** | **Brand**  **Name** | **Pharmaceutical Form** | **License** | **Batch** | **Manufacturer** |
| --- | --- | --- | --- | --- | --- |
| Generic | Inem | Ampules with imipenem monohydrate 500 mg powder + cilastatin sodium 500 mg + Na_2_CO_3_ | INVIMA 2004M-0003905 | 901766A, 712895A, 7128938 | IVAX Pharmaceuticals, Mexico SA de CV. Produced by license of Ranbaxy Laboratories Ltd., India |
| Innovator | Tienam | Ampules with imipenem monohydrate 500 mg powder + cilastatin sodium 500 mg + Na_2_CO_3_ | INVIMA 2007M-006539R1 | 936470, 7009Y, 5456X, 7230Y | Merck & Co. Inc., Elkton, VA, USA. Imported and marketed by FROSST Laboratories Inc., Bogota, Colombia |
