# Supplementary material for: Nontherapeutic equivalence of a generic product of imipenem-cilastatin is caused more by chemical instability of the active pharmaceutical ingredient (imipenem) than by its substandard amount of cilastatin
Source: PLoS One. 2019 Feb 6;14(2):e0211096. doi: 10.1371/journal.pone.0211096 (PMC6364906; doi:10.1371/journal.pone.0211096)
Supplement: S2 Table — (DOCX) [file pone.0211096.s002.docx]

**S2 Table. Determination of Minimal Inhibitory (MIC) and Bactericidal (MBC) Concentrations of One Generic and the Innovator Product of Imipenem-Cilastatin Against the Diverse Bacterial Strains Employed in the Study.**

| **Microorganism** | **Resistance**  **Phenotype** | **MIC [range] (mg/L)** | | | **MBC [range] (mg/L)** | | | **MBC/MIC ratio** | |
| --- | --- | --- | --- | --- | --- | --- | --- | --- | --- |
|  |  | Generic | Innovator | P_MIC_ | Generic | Innovator | P_MBC_ | Generic | Innovator |
| *S. aureus* GRP-0057 | Wild-type | 0.02 [*] | 0.02 [*] | 1.00 | 0.02 [*] | 0.02 [*] | 1.00 | 1.00 | 1.00 |
| *K. pneumoniae* GRP-0107 | Wild-type | 0.50 [*] | 0.50 [*] | 1.00 | 0.50 [*] | 0.50 [*] | 1.00 | 1.00 | 1.00 |
| *P. aeruginosa* GRP-0019 | Wild-type | 0.50 [*] | 0.50 [*] | 1.00 | 1.00 [*] | 1.00 [*] | 1.00 | 2.00 | 2.00 |
| *P. aeruginosa* GRP-0049 | MDR-CS† | 1.41 [1-2] | 1.19 [1-2] | 0.69 | 2.38 [2-4] | 2.38 [2-4] | 1.00 | 1.69 | 2.00 |
| *P. aeruginosa* GRP-0036 | MDR-CR¶ | 5.66 [4-8] | 8.00 [*] | 0.67 | 8.00 [*] | 16.0 [*] | 0.33 | 1.41 | 2.00 |
| *P. aeruginosa* ATCC 27853 | Wild-type | 0.84 [0.5-1] | 1.00 [*] | 0.69 | 1.41 [1-2] | 2.00 [1-4] | 0.69 | 1.68 | 2.00 |

**Abbreviations**: P_MIC_ = P-value for the comparison of MIC; P_MBC_ = P-value for the comparison of MBC

*No range; all values identical.

†MDR-CS: multi-drug resistant – carbapenem susceptible.

¶MDR-CR: multi-drug resistant – carbapenem resistant.
