# Supplementary material for: Nontherapeutic equivalence of a generic product of imipenem-cilastatin is caused more by chemical instability of the active pharmaceutical ingredient (imipenem) than by its substandard amount of cilastatin
Source: PLoS One. 2019 Feb 6;14(2):e0211096. doi: 10.1371/journal.pone.0211096 (PMC6364906; doi:10.1371/journal.pone.0211096)
Supplement: S3 Table — Comparison of one generic product of imipenem and the innovator (bioequivalence) in neutropenic mice infected in the thighs with P. aeruginosa GRP-0019. (DOCX) [file pone.0211096.s003.docx]

**S3 Table. Population Pharmacokinetics.**

| **Product** | **Number of support points** | **PK parameter** | **K_e_ (h^-1^)** | **V (L)** | **KCP (h^-1^)** | **KPC (h^-1^)** | **K_a_ (h^-1^)** |
| --- | --- | --- | --- | --- | --- | --- | --- |
| **Innovator** | 17 | **Median** | 4.47 | 0.01 | 20.6 | 7.92 | 9.31 |
|  |  | **Mean** | 5.09 | 0.01 | 21.6 | 13.5 | 9.85 |
|  |  | **SD** | 2.13 | 0.005 | 12.2 | 13.8 | 4.29 |
| **Generic** | 24 | **Median** | 4.82 | 0.01 | 12.5 | 4.03 | 8.86 |
|  |  | **Mean** | 4.81 | 0.01 | 10.1 | 5.01 | 7.67 |
|  |  | **SD** | 2.31 | 0.004 | 7.20 | 3.48 | 2.53 |

Comparison of one generic product of imipenem and the innovator (bioequivalence) in neutropenic mice infected in the thighs with *P. aeruginosa* GRP-0019.

**Abbreviations**: Ke, elimination rate constant; V, volume of distribution; KCP, first-order transfer rate constant from the central to peripheral compartment; KPC, first-order transfer rate constant from the peripheral to central compartment; Ka, absorption rate constant; SD, standard deviation.
