# Supplementary material for: Nontherapeutic equivalence of a generic product of imipenem-cilastatin is caused more by chemical instability of the active pharmaceutical ingredient (imipenem) than by its substandard amount of cilastatin
Source: PLoS One. 2019 Feb 6;14(2):e0211096. doi: 10.1371/journal.pone.0211096 (PMC6364906; doi:10.1371/journal.pone.0211096)
Supplement: S4 Table — Bacterial load of untreated controls at the time of the inoculum (h-2 or h-14) and when therapy with imipenem-cilastatin started (h0) and ended (h24), as well as bacterial growth (G) along the 24-hour treatment period, dose-range and dosing intervals designed for each infection model. (DOCX) [file pone.0211096.s004.docx]

**S4 Table. Basic Information for the Animal Models Employed in the Study.**

| **Thigh Model: Growth of *Staphylococcus aureus* GRP-0057 (log_10_ CFU/g) in five experiments** | | | | | | | | | | **Dose Range (mg/kg day)** | | **Dosing Interval (h)** |
| --- | --- | --- | --- | --- | --- | --- | --- | --- | --- | --- | --- | --- |
| **h-2*** | | **SD** | | **h0** | **SD** | **h24** | | **SD** | **G** | **Lowest** | **Highest** |  |
| 5.30 | | 0.03 | | 6.00 | 0.08 | 9.51 | | 0.33 | 3.51 | 0.3125 | 320 | 3 |
| 5.18 | | 0.10 | | 6.04 | 0.06 | 9.49 | | 0.16 | 3.45 | 0.3125 | 320 | 3 |
| 5.03 | | 0.07 | | 6.00 | 0.03 | 9.04 | | 0.12 | 3.04 | 0.6250 | 640 | 3 |
| 5.08 | | 0.02 | | 6.11 | 0.05 | 9.15 | | 0.05 | 3.04 | 0.6250 | 640 | 3 |
| ND | | ND | | 6.32 | 0.03 | 8.18 | | 0.04 | 1.86 | 0.3125 | 1280 | 3 |
| ***Lung Model: Growth of *Klebsiella pneumoniae* GRP-0107 (log_10_ CFU/g) in seven experiments** | | | | | | | | | | | | |
| 5.04 | | 0.06 | | 6.44 | 0.14 | 9.89 | | 0.17 | 3.44 | 10 | 320 | 3 |
| 5.45 | | 0.14 | | 6.57 | 0.15 | 9.96 | | 0.14 | 3.39 | 10 | 320 | 3 |
| 5.43 | | 0.30 | | 6.56 | 0.27 | 10.0 | | 0.03 | 3.46 | 10 | 320 | 3 |
| 5.29 | | 0.12 | | 6.41 | 0.42 | 9.82 | | 0.05 | 3.41 | 20 | 640 | 3 |
| 5.59 | | 0.22 | | 6.42 | 0.43 | 9.91 | | 0.17 | 3.48 | 10 | 1280 | 1 |
| 6.47 | | 0.36 | | 6.70 | 0.26 | 9.90 | | 0.18 | 3.20 | 10 | 1280 | 3 |
| 5.98 | | 0.01 | | 6.90 | 0.19 | 10.14 | | 0.10 | 3.24 | 10 | 1280 | 3 |
| **Brain Model: Growth of *Pseudomonas aeruginosa* GRP-0019 (log_10_ CFU/g) in five experiments** | | | | | | | | | | | | |
| ND | | ND | | 5.82 | 0.12 | 8.99 | | 0.45 | 3.17 | 20 | 320 | 3 |
| 4.02 | | 0.05 | | 4.89 | 0.24 | 10.2 | | 0.16 | 5.34 | 40 | 640 | 3 |
| 4.99 | | 0.07 | | 5.81 | 0.07 | 9.77 | | 0.35 | 3.97 | 40 | 640 | 3 |
| 6.07 | | 0.02 | | 6.68 | 0.12 | 9.52 | | 0.37 | 2.84 | 40 | 640 | 3 |
| 6.03 | | 0.03 | | 6.73 | 0.09 | 9.91 | | 0.36 | 3.18 | 40 | 640 | 3 |
| **Brain Model: Growth of *Pseudomonas aeruginosa* ATCC 27853 (log_10_ CFU/g) in a single experiment** | | | | | | | | | | | | |
| 4.28 | | 0.11 | | 4.71 | 0.13 | 9.63 | | 0.14 | 4.92 | 40 | 640 | 3 |
| **Brain Model: Growth of *Pseudomonas aeruginosa* GRP-0049 (log_10_ CFU/g) in two experiments** | | | | | | | | | | | | |
| 3.67 | | 0.27 | | 4.09 | 0.17 | 8.55 | | 0.46 | 4.46 | 40 | 640 | 3 |
| 4.13 | | 0.09 | | 4.33 | 0.11 | 8.88 | | 0.07 | 4.55 | 20 | 1280 | 3 |
| **Brain Model: Growth of *Pseudomonas aeruginosa* GRP-0036 (log_10_ CFU/g) in two experiments** | | | | | | | | | | | | |
| 3.88 | | 0.08 | | 4.71 | 0.10 | 9.61 | | 0.07 | 4.90 | 40 | 640 | 3 |
| 4.75 | | 0.11 | | 5.01 | 0.22 | 9.12 | | 0.74 | 4.11 | 160 | 2560 | 1 |
|  |  | |  | | | |  |  |  |  |  |  |

Bacterial load of untreated controls at the time of the inoculum (h-2 or h-14) and when therapy with imipenem-cilastatin started (h0) and ended (h24), as well as bacterial growth (G) along the 24-hour treatment period, dose-range and dosing intervals designed for each infection model.
