# Supplementary material for: Nontherapeutic equivalence of a generic product of imipenem-cilastatin is caused more by chemical instability of the active pharmaceutical ingredient (imipenem) than by its substandard amount of cilastatin
Source: PLoS One. 2019 Feb 6;14(2):e0211096. doi: 10.1371/journal.pone.0211096 (PMC6364906; doi:10.1371/journal.pone.0211096)
Supplement: S5 Table — Primary pharmacodynamic parameters (Emax, ED50, N) obtained by nonlinear regression, regression diagnostics, and statistical comparison by curve fitting analysis of the dose-response data from a generic product and the innovator of imipenem-cilastatin in diverse animal models of infection. (DOCX) [file pone.0211096.s005.docx]

**S5 Table. Pharmacodynamic Parameters, Regression Diagnostics and Statistical Analysis.**

| **Pathogen (Model)** | **Imipenem Product** | ***E_max_*** | **SEM** | ***ED_50_*** | **SEM** | ***N*** | **SEM** | **Multicol (PDP)** | **Best Fit Model** | **cAIC (%)** | **AdjR^2^** | **S_y\|x_** | **N/H** | **NLR P (CFA)** |
| --- | --- | --- | --- | --- | --- | --- | --- | --- | --- | --- | --- | --- | --- | --- |
| *S. aureus* GRP-0057 (Thigh) | Generic | 6.71 | 0.18 | 1.74 | 0.21 | 1.14 | 0.14 | None | Hill |  | 0.98 | 0.35 | OK | 0.0341 |
|  | Innovator | 6.83 | 0.22 | 1.11 | 0.18 | 0.87 | 0.13 | None | Hill |  | 0.96 | 0.39 | OK |  |
|  | Generic | 6.10 | 0.10 | 0.77 | 0.08 | 1.29 | 0.16 | None | Hill |  | 0.97 | 0.31 | OK | NA |
|  | Innovator | 6.93 | 0.16 | 0.75 | 0.11 | 0.78 | 0.10 | None | Hill |  | 0.96 | 0.33 | H(-) |  |
|  | Generic | 5.14 | 0.12 | 0.79 | 0.09 | 2.93 | 1.15 | None | Hill |  | 0.94 | 0.33 | OK | 0.0029 |
|  | Innovator | 5.93 | 0.13 | 1.09 | 0.12 | 1.63 | 0.24 | None | Hill |  | 0.96 | 0.35 | OK |  |
|  | Generic | 5.30 | 0.15 | 0.81 | 0.12 | 2.43 | 0.95 | None | Hill |  | 0.84 | 0.51 | H(-) | NA |
|  | Innovator | 6.12 | 0.10 | 0.91 | 0.09 | 1.49 | 0.22 | None | Hill |  | 0.94 | 0.33 | OK |  |
|  | Generic | 5.73 | 0.09 | 1.13 | 0.10 | 1.54 | 0.20 | None | Hill |  | 0.86 | 0.58 | OK | <0.0001 |
|  | Innovator | 6.32 | 0.09 | 0.96 | 0.08 | 1.01 | 0.10 | None | Hill |  | 0.89 | 0.49 | OK |  |
|  | Generic | 4.84 | 0.07 | 1.49 | 0.15 | 1.40 | 0.18 | None | Hill |  | 0.96 | 0.27 | OK | <0.0001 |
|  | Innovator | 5.22 | 0.06 | 0.89 | 0.09 | 1.15 | 0.11 | None | Hill |  | 0.97 | 0.22 | OK |  |
| *K. pneumoniae* GRP-0107 (Lung) | Generic | 7.33 | 0.16 | 1.38 | 0.03 | 1.00 | 0.06 | None | Gauss | 99.57 | 0.96 | 0.44 | OK | 0.629 |
|  | Innovator | 6.98 | 0.29 | 1.33 | 0.06 | 1.16 | 0.15 | None | Gauss | 52.01 | 0.85 | 0.79 | OK |  |
|  | Generic | 7.04 | 0.20 | 1.38 | 0.04 | 1.02 | 0.08 | None | Gauss | 54.38 | 0.94 | 0.53 | OK | Different Equation |
|  | Innovator | 7.51 | 0.47 | 26.8 | 4.33 | 1.14 | 0.19 | *E_max_, ED_50_* | Hill | 60.44 | 0.93 | 0.51 | OK |  |
|  | Generic | 6.75 | 0.16 | 1.52 | 0.03 | 0.91 | 0.05 | None | Gauss | 58.05 | 0.97 | 0.42 | OK | Different Equation |
|  | Innovator | 6.60 | 0.25 | 28.0 | 2.57 | 1.50 | 0.19 | None | Hill | 90.6 | 0.96 | 0.40 | OK |  |
|  | Generic | 6.64 | 0.19 | 1.56 | 0.04 | 0.99 | 0.06 | None | Gauss | 95.79 | 0.92 | 0.49 | OK | Different Equation |
|  | Innovator | 6.58 | 0.23 | 29.0 | 2.61 | 1.90 | 0.36 | None | Hill | 98.81 | 0.90 | 0.56 | OK |  |
|  | Generic | 5.80 | 0.15 | 29.8 | 2.39 | 1.90 | 0.26 | None | Hill | 99.16 | 0.94 | 0.47 | OK | 0.0408 |
|  | Innovator | 5.81 | 0.13 | 22.6 | 1.57 | 1.77 | 0.21 | None | Hill | >99.99 | 0.95 | 0.40 | OK |  |
|  | Generic | 5.39 | 0.12 | 33.5 | 1.90 | 2.98 | 0.44 | None | Hill | >99.99 | 0.96 | 0.41 | OK | 0.0538 |
|  | Innovator | 5.43 | 0.15 | 26.4 | 2.15 | 2.34 | 0.39 | None | Hill | >99.99 | 0.92 | 0.51 | OK |  |
|  | Generic | 6.14 | 0.19 | 1.68 | 0.05 | 1.19 | 0.08 | None | Gauss | 97.81 | 0.96 | 0.42 | OK | Different Equation |
|  | Innovator | 5.61 | 0.16 | 22.5 | 2.60 | 2.61 | 0.44 | None | Hill | >99.99 | 0.95 | 0.47 | OK |  |
|  | Generic | 6.67 | 0.13 | 1.49 | 0.03 | 1.10 | 0.04 | None | Gauss | 99.8 | 0.85 | 0.81 | OK | Different Equation |
|  | Innovator | 6.22 | 0.12 | 23.8 | 1.33 | 1.81 | 0.17 | None | Hill | >99.99 | 0.85 | 0.75 | OK |  |
| *P. aeruginosa* GRP-0019 (Brain) | Generic | 5.33 | 0.19 | 82.9 | 3.82 | 3.50 | 0.57 | None | Hill |  | 0.98 | 0.31 | OK | 0.697 |
|  | Innovator | 5.73 | 0.40 | 86.6 | 8.90 | 2.57 | 0.54 | None | Hill |  | 0.95 | 0.51 | OK |  |
|  | Generic | 7.43 | 0.35 | 102.9 | 9.15 | 2.13 | 0.34 | None | Hill |  | 0.95 | 0.56 | OK | 0.282 |
|  | Innovator | 7.36 | 0.21 | 87.2 | 4.78 | 2.15 | 0.22 | None | Hill |  | 0.98 | 0.36 | OK |  |
|  | Generic | 6.05 | 0.28 | 99.9 | 7.75 | 3.82 | 0.88 | None | Hill |  | 0.93 | 0.63 | OK | 0.5 |
|  | Innovator | 6.12 | 0.23 | 88.8 | 5.83 | 3.09 | 0.57 | None | Hill |  | 0.95 | 0.50 | OK |  |
|  | Generic | 4.68 | 0.23 | 103.9 | 13.8 | NS | ∞ | None | Hill |  | 0.94 | 0.55 | OK | NA |
|  | Innovator | 4.84 | 0.22 | NS | ∞ | NS | ∞ | *ED_50_, N* | Hill |  | 0.94 | 0.54 | H(-) |  |
|  | Generic | 5.13 | 0.22 | 102.3 | 8.25 | 5.56 | 1.52 | None | Hill |  | 0.93 | 0.53 | H(-) | NA |
|  | Innovator | 5.35 | 0.26 | 103.9 | 8.71 | 4.66 | 1.18 | None | Hill |  | 0.91 | 0.61 | H(-) |  |
| *P. aeruginosa* GRP-0049 (Brain) | Generic | 5.27 | 0.58 | 101.5 | 22.2 | 1.64 | 0.49 | *E_max_* | Hill |  | 0.85 | 0.67 | OK | NA |
|  | Innovator | 5.33 | 0.31 | 93.7 | 10.5 | 1.91 | 0.35 | None | Hill |  | 0.94 | 0.45 | OK |  |
|  | Generic | 5.52 | 0.15 | 53.0 | 3.38 | 3.70 | 0.65 | None | Hill |  | 0.95 | 0.50 | OK | 0.0059 |
|  | Innovator | 6.43 | 0.20 | 62.5 | 4.28 | 3.42 | 0.64 | None | Hill |  | 0.94 | 0.62 | OK |  |
| *P. aeruginosa* GRP-0036 (Brain) | Generic | 5.53 | 0.43 | 261.9 | 23.3 | 3.08 | 0.61 | None | Hill |  | 0.96 | 0.44 | OK | NA |
|  | Innovator | 5.92 | 0.96 | 224.4 | 59.6 | 1.69 | 0.43 | *E_max_, ED_50_* | Hill |  | 0.92 | 0.53 | H(-) |  |
|  | Generic | 6.05 | 0.96 | 598.1 | 180.7 | 1.52 | 0.47 | *E_max_, ED_50_* | Hill |  | 0.87 | 0.72 | OK | NA |
|  | Innovator | 7.39 | 1.98 | NS | ∞ | 1.16 | 0.34 | All | Hill |  | 0.90 | 0.58 | H(-) |  |
| *P. aeruginosa* ATCC 27853 (Brain) | Generic | 6.70 | 0.61 | 152.3 | 23.9 | 1.92 | 0.42 | None | Hill |  | 0.93 | 0.63 | OK | <0.0001 |
|  | Innovator | 6.38 | 0.25 | 85.7 | 6.17 | 2.85 | 0.53 | None | Hill |  | 0.94 | 0.54 | OK |  |

Primary pharmacodynamic parameters (*E_max_, ED_50_, N*) obtained by nonlinear regression, regression diagnostics, and statistical comparison by curve fitting analysis of the dose-response data from a generic product and the innovator of imipenem-cilastatin in diverse animal models of infection.

**Abbreviations**: *E_max_*, maximal effect in Δlog_10_ CFU/g at 24h; SEM, standard error of the mean; *ED_50_*, Effective Dose to reach 50% of the *E_max_* in mg/kg per day; *N*, Hill’s slope; Multicol. (PDP), pharmacodynamic parameter affected by multicollinearity; cAIC, corrected Akaike’s information criteria expressed as a percentage; AdjR^2^, adjusted coefficient of determination; S_y|x_, standard error of the estimate; N/H, Normality/Homoscedasticity assumptions: OK implies no violation, H(-) implies homoscedasticity violation, normality was not violated; NLR P (CFA), P value for the comparison of both products nonlinear regressions by curve fitting analysis; NA, the comparison is not applicable because one or both products had problems with the regression diagnostics; ∞ infinite error implying that the PDP is not significantly different from zero.
